# A Week of Oral Terbinafine Pulse Regimen Every Three Months to Treat all Dermatophyte Onychomycosis

**DOI:** 10.3390/jof5030082

**Published:** 2019-09-04

**Authors:** Anarosa B. Sprenger, Katia Sheylla Malta Purim, Flávia Sprenger, Flávio Queiroz-Telles

**Affiliations:** 1 Santa Casa de Curitiba Hospital, Clinic of Diseases and Surgery of the Nail Apparatus, Department of Dermatology, Praça Rui Barbosa, 694, 80.010-030 Curitiba, Brazil; 2 Hospital de Clínicas de Curitiba—Universidade Federal do Paraná (UFPR), Clinic of Dermatology, Rua General Carneiro, 181, 80.060-900 Curitiba, Brazil; 3 Univerdidade Federal do Paraná (UFPR), Rua General Carneiro, 181, 80.060-900 Curitiba, Brazil; 4 Hospital de Clínicas de Curitiba—Paraná Federal University (UFPR), Department of Public Health, Rua General Carneiro, 181, 80.060-900 Curitiba, Brazil

**Keywords:** administration, allylamines/terbinafine, Arthrodermataceae/drug effects, drug compounding, humans, onychomycosis, oral, antifungal agents/administration, dosage/adverse effects/pharmacology

## Abstract

Terbinafine has proved to treat numerous fungal infections, including onychomycosis, successfully. Due to its liver metabolization and dependency on the cytochrome P450 enzyme complex, undesirable drug interaction are highly probable. Additionally to drug interactions, the treatment is long, rising the chances of the appearance of side effects and abandonment. Pharmacokinetic data suggest that terbinafine maintains a fungicidal effect within the nail up to 30 weeks after its last administration, which has aroused the possibility of a pulse therapy to reduce the side effects while treating onychomycosis. This study’s goal was to evaluate the effectiveness of three different oral terbinafine regimens in treating onychomycosis due to dermatophytes. Sixty-three patients with onychomycosis were sorted by convenience in three different groups. Patients from group 1 received the conventional terbinafine dose (250 mg per day for 3 months). Group 2 received a monthly week-long pulse-therapy dose (500 mg per day for 7 days a month, for 4 months) and group 3 received a 500 mg/day dose for 7 days every 3 months, totaling four treatments. There were no statistical differences regarding the effectiveness or side effects between the groups. Conclusion: A quarterly terbinafine pulse regimen can be a possible alternative for treating onychomycosis caused by dermatophytes.

## 1. Introduction

Onychomycosis due to dermatophytes, yeasts, and non-dermatophyte molds comprises 50% of all cases of nail disease [1]. Known individual risk factors for its development are nail trauma, age, smoking, immunosuppression, obesity, psoriasis, and other causes of onychodystrophy, peripheral arterial disease, and diabetes mellitus [2,3,4,5,6]. Male patients have been reported to have more severe and chronic onychomycosis [7].

The susceptibility to onychomycosis is inherited and it is often observed among family members. Some studies revealed the existence of polymorphisms in genes of the major histocompatibility complex related to higher susceptibility to onychomycosis from dermatophytes, particularly haplotypes HLA-DR8 and HLA-DR1 [8,9,10,11]

The estimated global prevalence of onychomycosis is 5.5% [12]. More than 60% of these infections are caused by dermatophytes, mainly *Trichophyton rubrum, Trichophyton mentagrophytes, Epidermophyton floccosum*, and *Microsporum spp*. The remaining infections can be due non-dermatophyte molds, predominantly *Scopulariopsis brevicaulis, Aspergillus spp., Acremonium, Fusarium sp.p, Alternaria alternata,* and *Neoscytalidium spp.*, or to yeasts, such as *Candida albicans* [13].

Trauma caused by shoes produces toenail changes, especially in people with orthopedic changes that cause faulty poor adaptation of the feet in shoes, that are identical to some onychomycosis at the time of clinical presentation [14]. Most toenails abnormalities are, in fact, due to the pressure exerted by shoes and not by fungi. It has already been observed that the presence of non-dermatophyte molds in a dystrophic nail could be considered a secondary niche of colonization in a nail previously damaged by trauma, rather than onychomycosis [15].

Aside from the high prevalence, onychomycosis has therapeutic challenges. The available therapeutic arsenal is not vast, and there are high rates of resistance and recurrence, making it a noteworthy public health issue [16,17].

Topical and oral antifungals are the treatment options for onychomycosis. Topical therapy is used in children and adults with mild to moderate onychomycosis or for single affected digits. Ciclopirox and amorolfine are the most used topical agents, and recently, tavaborole and efinaconazole have been introduced in North America [18,19,20].

FDA-approved oral treatment for onychomycosis includes terbinafine and itraconazole, and fluconazole is used off-label. Due to fewer collateral effects and higher cure rates, terbinafine is usually preferred over itraconazole [21]. The standard dosage is 250 mg per day for 6 weeks for fingernails or 12 weeks for toenails. [22]. Some pharmacokinetic studies have shown that terbinafine can be detected in the nail plate in concentrations above the minimal inhibitory concentrations for dermatophytes and other fungi 36 weeks post-treatment [23,24,25]. Pulse regimens have been proposed to reduce the side effects and risks of interaction with other medication. Most studies have shown a superior efficacy of terbinafine compared to itraconazole pulse regimens and similar efficacy compared to a conventional terbinafine dose [26,27].

The standard dose for a terbinafine pulse regimen is 500 mg per day for 7 days a month, twice or three times for fingernails and three to four times for toenails [28].

Zaias and Rebell [29] have described considerable terbinafine efficacy utilizing a quarterly pulse therapy regimen for the treatment of distal subungual onychomycosis (DSO) caused by *T. rubrum.*

We performed an open non-randomized study in which standard terbinafine regimens were compared with a pulse terbinafine regimen of 500 mg/day for 7 days every 3 months to treat onychomycosis caused by dermatophytes.

## 2. Materials and Methods

### 2.1. Patients

In total, we included 63 patients (34 women, 29 men) aged between 24 and 70 years who had visited the Dermatology Outpatient’s Clinic at Santa Casa de Curitiba Hospital between August 2013 and July 2016. Those aged 18 years or older and diagnosed with dermatophyte onychomycosis based on clinical manifestations and confirmed using mycological culture were eligible to participate. Patients with less than 25% of the nail affected by the disease, liver or kidney impairment, pregnant or lactating were excluded. All participants signed an informed consent term.

Two measurements were taken of infected nails before, during the appointments, and at the end of each group treatment. The first one was the length of the nail plate from the free edge to the proximal nail fold, and the second one was the length of the visible fungal-infected portion. The percentage of the compromised nail was then calculated.

Data concerning age, sex, occupation, sport activities, comorbidities, and concomitant use of medications were recorded. Patients were divided into three groups, according to their order of attendance.

In Group 1, 20 patients received continuous 250 mg terbinafine for 3 months. In Group 2, 21 patients received a terbinafine 500 mg monthly pulse regimen, for 4 months. In Group 3, 22 patients received terbinafine 500 mg/day for 7 days, every 3 months and completed four pulse regimens.

Patients of Group 1 were asked to attend the hospital monthly. Patients of Group 2 had appointments every 2 months. Patients of Group 3 attended the hospital every 3 months. During the appointments, all patients were examined, questioned concerning any possible side effects, and received a new supply of terbinafine containing 28 tablets.

Mycological cultures were provided for all patients who completed the study.

### 2.2. Evaluation of Therapeutic Response

The degrees of improvement were classified as follows: total cure (TC), clinical disease-free nail and a negative mycological culture; mycological cure (MC), <25% of nail impairment and a negative mycological culture; clinical improvement (CI), <25% of nail impairment and a positive mycological culture; therapeutic failure (TF), unchanged clinical examination or worsening and a positive mycological culture.

### 2.3. Statistical Methodology

For the quantitative variables, a comparison between treatments was undertaken using a Kruskal–Wallis non-parametric test, suitable for the analysis of independent samples and variables with interval measurements without normal distribution [30]. For the group comparisons, in relation to the categorical variables, a non-parametric chi-square test was applied. In all tests, a *p* value of 5% was considered statistically significant.

### 2.4. Demographic Characteristics

Table 1 shows the patients’ demographic characteristics. A possible occupational relationship refers to occupations that may lead to greater exposure to fungi on the feet or hands, such as those requiring the use of safety shoes and those in which patients had been exposed to humidity, heat, or trauma. Sport activities were also considered, as trauma is a relevant factor in the speed of growth of a nail plate and, therefore, in the recovery of infected nails. Gender, age, previous treatments, which are also relevant factors in a treatment response, were evaluated, as was the concomitant use of other medications that can interact with terbinafine [31].

The isolated fungi in the mycological cultures were *Trichophyton sp*, *T. rubrum*, *T. mentagrophytes*, and *Microsporum gypseum* (Table 2).

**Figure 1:**
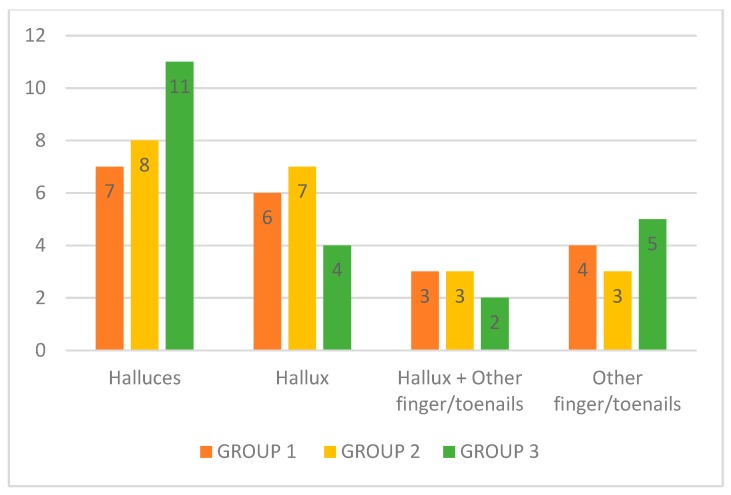
Toenails and fingernails affected (*n* = 63). Nail disease distribution according to groups before the treatment.

The hallux was the most affected nail (*n* = 43) in all three groups, followed by the 4th, 5th, and 3rd toenail and the thumbnail (*n* = 20). There was no significant difference among the groups regarding the distribution of affected fingers or toenails (Figure 1).

**Figure 2:**
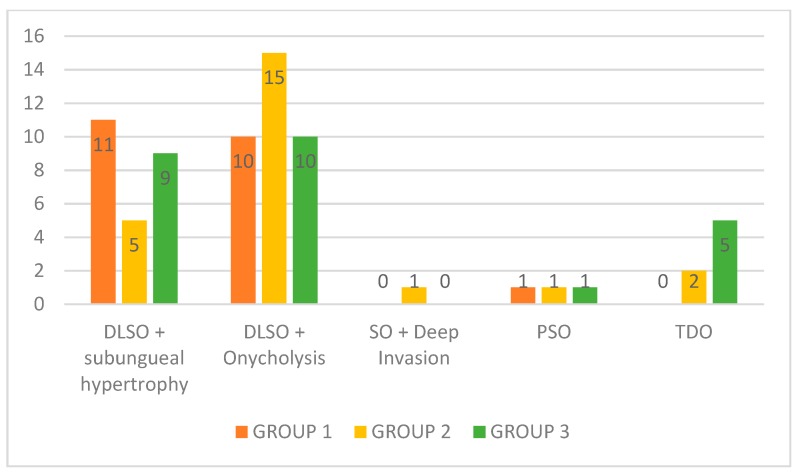
Clinical classification according to groups (*n* = 71). Clinical classification according to groups before the treatment. DLSO + subungual hypertrophy: distal lateral subungual onychomycosis with subungual hypertrophy; DLSO + Onycholysis: distal lateral subungual onychomycosis with onycholysis; SO + Deep Invasion: superficial onychomycosis with deep invasion; PSO: proximal subungual onychomycosis; TDO: total dystrophic onychomycosis.

According to the clinical classification proposed by Baran and Hay [32], most patients were identified with a distal lateral subungual onychomycosis (DLSO) with subungual onycholysis (*n* = 35), followed by DLSO with subungual hypertrophy (*n* = 25), total dystrophic onychomycosis (TDO) (*n* = 7), proximal subungual onychomycosis (PSO) (*n* = 3), and superficial onychomycosis (SO) with deep invasion (*n* = 1). Nine patients had more than one nail type of onychomycosis in different nails; therefore, for the clinical classification, the sample included 71 affected nails (Figure 2).

## 3. Results

**Figure 3:**
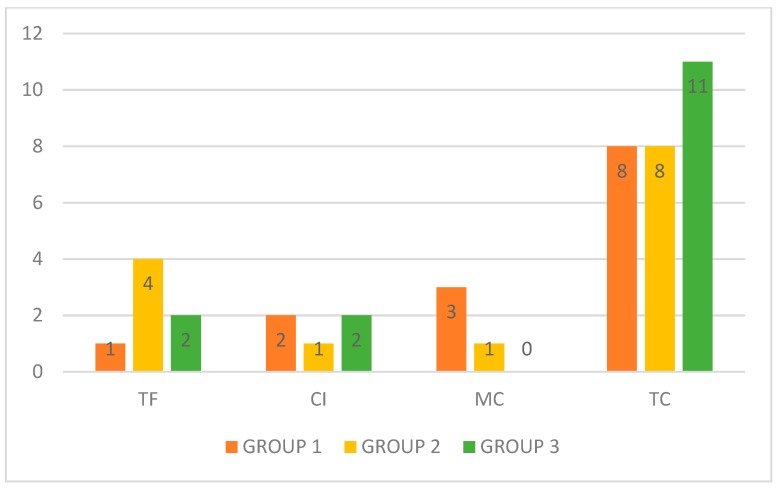
Response to terbinafine (*n* = 43). TF: therapeutic failure; CI: clinical improvement; MC: mycological cure; TC: total cure.

In total, 43 patients completed the study comprising 14, 14, and 15 patients in Groups 1, 2, and 3, respectively. Thirteen (92.86%), 10 (71.43%), and 13 (86.67%) patients from groups 1, 2, and 3, respectively, presented with TC, MC, or CI (Figure 3).

After applying the chi-square test, no significant difference was observed between the groups (*p* = 0.280) concerning the response to terbinafine. However, among the patients who finished the study, the majority (83.72%, *p* = 0.001) showed at least one degree of improvement (TC, MC, or CI).

### 3.1. Relationship between Treatment Results, Affected Nails, Clinical Classification, Presence of Comorbidities, Use of Medications, and Isolated Fungi

Table 3 shows clinical and microbiological data, such as treatment results, affected nails, clinical classification, presence of comorbidities, use of medications, and isolated fungi for all participants that finished the study.

### 3.2. Dropouts and Side Effects

Twenty patients did not complete the study, five (7.93%) of them because of side effects. The other 15 (23.90%) patients dropped out for personal reasons.

The most observed side effects were gastralgia (Group 2, *n* = 4, Group 3, *n* = 1) and cutaneous rash (Group 1, *n* = 1). None of these patients had comorbidities or used medications that could interact with terbinafine. (Table 4).

## 4. Discussion

Since its introduction, terbinafine has been considered more effective than other antifungals available to treat dermatophytosis [33,34,35,36].

Undesirable side effects have been associated with terbinafine use, especially during a long treatment period, including gastrointestinal side effects, cutaneous rash, headache, myalgia, and, rarely, hepatotoxicity, drug-induced lupus erythematosus, Sjogren’s syndrome, Stevens–Johnson syndrome, toxic epidermal necrolysis, alopecia, and psoriasis [37,38,39,40].

Terbinafine is metabolized in part by the cytochrome P450 isoenzymes, particularly CYP2D6, which explains the lower rates of drug interactions in comparison with other anti-fungal agents [41,42]. Terbinafine is contraindicated in patients with allergy to terbinafine or in patients with liver dysfunction and it may be used with caution with selective serotonin reuptake inhibitors, C1 antiarrhythmics, and monoaminoxidase inhibitors [43,44]. Seven patients that completed this study used antidepressants, six of them presented with TC, and one with MC.

Some studies have shown terbinafine presence in nails in concentrations above the minimal inhibitory concentrations of 0.0015–0.01 mg/ml for dermatophytes and 0.06–0.025 mg/ml for other fungi, e.g., *Aspergillus* species, for more than 36 weeks post-treatment and high plasmatic levels 12 weeks after the beginning of treatment [16,20,25,33,45]. The use of terbinafine for onychomycosis has been compared with that of other anti-fungal agents, especially itraconazole, or even with terbinafine itself in different types of regimens associated or not with a topical treatment. Most studies have focused on the administration of terbinafine doses between 250 and 500 mg per day for 3 or 4 months or on intermittent therapy involving 4 weeks of terbinafine followed by a 4-week period off terbinafine, and then additional 4 weeks of terbinafine treatment [46,47].

This study aimed to determine whether a longer drug interval period could result in effectiveness rates similar to or higher than those described for other regimens (of approximately 57%) in previous studies [48,49] and also if the proposed regimen can be more economical. The demographic variables in our study were similar to those in other terbinafine comparative studies [7,28,50]. The three groups showed similar TC, MC, and CI rates.

Compared to the Zaias and Rebell’s study [29], which described efficacy using a 250 mg quarterly terbinafine regimen pulse for onychomycosis caused by T. *rubrum*, in our study, two patients of Group 3 (13.33%) had TC and one patient (6.66%) had clinical improvement of onychomycosis caused by *T. mentagrophytes.* We opted for the 500 mg quarterly dose in order to compare the efficacy with that of the standard 500 mg monthly dose used in most of the published studies based on pulse regimens. In addition, this is the terbinafine dose of pulse regimen utilized in Brazil. Further studies with a more significant number of participants are necessary to compare the effectiveness of the trimester schemes of oral terbinafine in the treatment of all types of dermatophyte onychomycosis.

Treatment outcomes for onychomycosis can also vary according to age, clinical presentation, comorbidities, and the use of medications that may interact with antifungal treatments [32,51,52]. DLSO with hypertrophy with or without dermatophytoma, PSO, and TDO can be more resistant to treatment. SO may be difficult to treat if associated with immunodeficiency [53,54]. This study did not reveal significant differences between the outcomes and the clinical presentation, the presence of comorbidities, or the concomitant use of medications, probably because of the limited number of participants.

The asymmetric gait nail unit syndrome (AGNUS), firstly described by Zaias et al. in 2012, is caused by repetitive toe trauma in a closed shoe in patients with asymmetric walking due to orthopedic abnormalities. The resulting nail changes can undoubtedly be clinically identical to onychomycosis [55]. In our study, the possible orthopedic abnormalities were not evaluated, but cultures were performed before and after the treatment, confirming that all participants had onychomycosis. In future onychomycosis studies, the evaluation of the concomitant presence of AGNUS can be useful, since it can change the clinical classification and the cure criteria.

As life expectancy is increasing globally, it is common for patients with onychomycosis to present with comorbidities and take more than one long-term medication [36,56,57,58,59]. As the speed of nail growth decreases with age, terbinafine is a good treatment option because it can enhance the speed of growth in the nail plate; therefore, the portion of compromised nail plate grows faster to the free edge and can be eliminated through cutting [58,60,61]. All groups in this study comprised some patients with comorbidities. The most common were depression (*n* = 7), obesity (*n* = 5), hypothyroidism (*n* = 3), diabetes (*n* = 1), and HIV active infection (*n* = 1). Only one patient with a comorbidity, who had hypothyroidism, presented with TF.

The pulse regimen therapy (groups 2 and 3) had the lowest cost of treatment. Group 1 used a total of 84 tablets, while groups 2 and 3 used 56 tablets each. The quarterly pulse regimen had the same cost compared to the conventional terbinafine pulse regimen but might be more financially appealing since patients are required to purchase a second terbinafine supply only six months after the start of treatment [48,62,63].

The long drug rest interval may compromise adherence to treatment due to patients forgetting the quarterly dose, which is an issue that can be addressed through counseling patients, family members responsible for them, or, eventually, caregivers of elderly persons using a simple reminder message recorded on their cell phone [64].

## 5. Conclusions

Despite the limited sample, a pulse therapy regimen using terbinafine 500 mg per day for a week every three months was found to be a potentially useful alternative in the treatment of onychomycosis by dermatophytes. Further studies involving a more significant number of patients are necessary to confirm the effectiveness of this treatment regimen.

## Figures and Tables

**Table 1:** Demographic characteristics (*n* = 63).

Demographic Characteristics	Group 1	Group 2	Group 3	Total
**1. Age**				
Average	47	48	48.27	47.78
n	20	21	22	63
Minimum	26	27	24	24
Maximum	67	70	70	70
**2. Sex**				
Female (%)	12 (60.00%)	12 (57.14%)	10 (45.45%)	34
Male (%)	8 (40.00%)	9 (42.86%)	12 (54.55%)	29
Total	20	21	22	63
**3. Occupation (%)**				
Possible occupational relationship	6 (30.00%)	9 (42.86%)	5 (22.73%)	20
No possible occupational relationship	14 (70.00%)	12 (57.14%)	17 (77.72%)	43
Total	20	21	22	63
**4. Previous Treatment**				
No (%)	18 (90.00%)	19 (90.48%)	22 (100%)	59
Yes (%)	2 (10.00%)	2 (9.52%)	0 (0.00%)	4
Total	20	21	22	63
**5. Sports Activities (%)**				
None (%)	9 (45.00%)	17 (80.95%)	17 (77.27%)	43
Effect on the feet (%)	2 (10.00%)	2 (9.52%)	0 (0.00%)	4
No effect on the feet (%)	9 (45.00%)	2 (9.52%)	5 (22.73%)	16
Total	20	21	22	63
**6. Use of Medicines**				
No interaction (%)	14 (70.00%)	19 (90.48%)	18 (81.82%)	51
Antidepressants (%)	4 (20.00%)	1 (4.76%)	1 (4.55%)	6
Beta-blockers (%)	1 (5.00%)	0 (0.00%)	1 (4.55%)	2
Immunosuppressants (%)	1 (5.00%)	0 (0.00%)	0 (0.00%)	1
>1 Possible interaction (%)	0 (0.00%)	1 (4.76%)	2 (9.09%)	3
Total	20	21	22	63
**7. Comorbidities**				
None (%)	13 (65.00%)	15 (71.43%)	16 (72.73%)	44
Diabetes (%)	0 (0.00%)	0 (0.00%)	1 (4.55%)	1
Obesity (%)	1 (5.00%)	1 (4.76%)	1 (4.55%)	3
Hypothyroidism (%)	2 (10.00%)	1 (4.76%)	0 (0.00%)	3
Depression (%)	2 (10.00%)	3 (14.29%)	1 (4.55%)	6
Immunodeficiency (%)	1 (5.00%)	1 (4.76%)	1 (4.55%)	3
>1 Comorbidities (%)	1 (5.00%)	0 (0.00%)	2 (9.09%)	3
Total	20	21	22	63

**Table 2:** Isolated fungi (*n* = 63).

Fungus	Group 1	Group 2	Group 3	Total
*Trichophyton sp* (n, %)	15 (75.00%)	13 (61.90%)	12 (54.55%)	40
*Trichophyton mentagrophytes* (n, %)	2 (10.00%)	4 (19.05%)	5 (22.73%)	11
*Trichophyton rubrum* (n, %)	3 (15.00%)	4 (19.05%)	4 (18.18%)	11
*Microsporum gypseum* (n, %)	0 (0.00%)	0 (0.00%)	1 (4.55%)	1
Total	20	21	22	63

**Table 3:** Treatment results, affected nails, clinical classification, comorbidities, use of medications, and isolated fungi. TC: total cure, MC: mycological cure, CI: clinical improvement, TF: therapeutic failure.

Result	Fingernails/Toenails	Clinical Calssification	Comorbidities	Medications	Isolated Fungi
TC	Right hallux	DLSO + onycholysis	0	0	*Trichophyton sp*
TC	Right hallux	DLSO + hypertrophy	0	0	*Trichophyton sp*
TC	Right hallux + 4rth left toenail	DLSO + hypertrophy	0	0	*Trichophyton sp*
TC	Halluces	DLSO + hypertrophy	depression	antidepressant	*Trichophyton sp*
TC	Right hallux	DLSO + hypertrophy	depression	antidepressant	*Trichophyton sp*
TC	Halluces	DLSO + onycholysis	0	0	*T. rubrum*
TC	Right hallux	DLSO + hypertrophy	0	0	*T. mentagrophytes*
TC	Left hallux	DLSO + onycholysis	hypothyroidism	0	*T. rubrum*
MC	Halluces	DLSO + onycholysis	obesity + depression	antidepressant	*Trichophyton sp*
MC	Right hallux	DLSO + onycholysis	0	0	*T. mentagrophytes*
MC	Halluces	DLSO + onycholysis	0	0	*Trichophyton sp*
CI	Halluces	DLSO + onycholysis	0	0	*Trichophyton sp*
CI	Halluces	DLSO + onycholysis	0	0	*Trichophyton rubrum*
TF	Right hallux	DLSO + hypertrophy	hypothyroidism	0	*T. rubrum*
TC	Right hallux	DLSO + onycholysis	depression	0	*T. mentagrophytes*
TC	Right hallux	DLSO + onycholysis	hypothyroidism	0	*Trichophyton sp*
TC	4rth Right fingernail	DLSO + onycholysis	0	0	*Trichophyton sp*
TC	Halluces	DLSO + hypertrophy	0	0	*Trichophyton sp*
TC	Halluces	DLSO + onycholysis	0	0	*T. rubrum*
TC	Halluces	DLSO + onycholysis	depression	antidepressant	*Trichophyton sp*
TC	Left hallux	DLSO + hypertrophy	0	0	*Trichophyton sp*
TC	Right hallux	DLSO + onycholysis	HIV	antiretrovirals	*Trichophyton sp*
MC	Left hallux	DLSO + onycholysis	0	0	*Trichophyton sp*
CI	Halluces	DLSO + onycholysis	0	0	*T. mentagrophytes*
TF	Right hallux + 2nd left toenail	DLSO + onycholysis	0	0	*T. rubrum*
TF	Halluces	PSO + SO	0	0	*Trichophyton sp*
TF	Halluces	DLSO + hypertrophy	0	0	*T. mentagrophytes*
TF	Halluces	DLSO + hypertrophy + DLSO + onycholysis	0	0	*Trichophyton sp*
TC	Right hallux + 3rd left toenail	DLSO + hypertrophy	depression	antidepressant	*T. mentagrophytes*
TC	2nd right + 3rd left toenails	TDO	0	0	*Trichophyton sp*
TC	Right hallux	TDO	obesity	0	*Trichophyton sp*
TC	Right hallux	DLSO + onycholysis	0	0	*Trichophyton sp*
TC	2nd right toenail	DLSO + onycholysis + TDO	obesity + depression	antidepressant	*T. rubrum*
TC	Left hallux	DLSO + hypertrophy	0	0	*Trichophyton sp*
TC	Left hallux	DLSO + onycholysis + TDO	diabetes	o	*Trichophyton sp*
TC	Right hallux	DLSO + onycholysis	0	0	*Trichophyton sp*
TC	Halluces	DLSO + onycholysis	0	0	*T. rubrum*
TC	2nd right + 2nd left toenails	TDO	0	0	*Trichophyton sp*
TC	2nd right toenail	DLSO + hypertrophy	0	0	*Trichophyton mentagrophytes*
CI	Halluces	DLSO + onycholysis	0	0	*Trichophyton sp*
CI	3rd right + 3rd left toenail	DLSO + hypertrophy	0	0	*T. mentagrophytes*
TF	Halluces	DLSO + onycholysis	0	0	*Trichophyton sp*
TF	Halluces	DLSO + onycholysis	0	0	*T. mentagrophytes*

There was no significant difference in the treatment results concerning the affected nails (*p* = 0.750), clinical classification (*p* = 0.580), presence of comorbidities (*p* = 0.730), use of medications (*p* = 0.660), and the isolated fungi (*p* = 0.770).

**Table 4:** Dropouts and side effects.

Side Effects/Dropout	Group 1	Group 2	Group 3	Total
None	14 (70.00%)	14 (66.67%)	15 (68.18%)	43
Gastralgia	0 (0.00%)	3 (14.28%)	1 (4.55%)	4
Cutaneous rash	1 (5.00%)	0 (0.00%)	0 (0.00%)	1
Did not complete (personal reasons)	5 (25.00%)	4 (19.05%)	6 (27.27%)	15
Total	20	21	22	63

## Abbreviations

CI	clinical improvement
DLSO	distal lateral subungual onychomycosis
MC	mycological cure
SO	superficial onychomycosis
TC	total cure
TDO	total dystrophic onychomycosis
TF	therapeutic failure
AGNUS	asymmetric gait nail unit syndrome
